# Role of *p*-Benzoquinone in the Photocatalytic Production of Solketal

**DOI:** 10.3390/molecules30163339

**Published:** 2025-08-11

**Authors:** Alejandro Ariza-Pérez, Juan Martín-Gómez, M. Carmen Herrera-Beurnio, Francisco J. López-Tenllado, Jesús Hidalgo-Carrillo, Alberto Marinas, Francisco J. Urbano

**Affiliations:** Departamento de Química Orgánica, Instituto Químico para la Energía y el Medioambiente (IQUEMA), Universidad de Córdoba, E-14071 Córdoba, Spain; q82arpea@uco.es (A.A.-P.); b52hebem@uco.es (M.C.H.-B.); b42lotef@uco.es (F.J.L.-T.); jesus.hidalgo@uco.es (J.H.-C.); alberto.marinas@uco.es (A.M.)

**Keywords:** photocatalysis, solketal synthesis, *p*-benzoquinone, electron paramagnetic resonance spin-trapping experiments, photoacid, excited-state proton transfer process, hydrogen atom transfer process, TiO_2_

## Abstract

The role of *p*-benzoquinone (BQ) as a photocatalyst in the synthesis of solketal under UV irradiation has been studied, along with the combined use of BQ/TiO_2_ P25 as a photocatalytic system for the process. The presence of the O_2_/O_2_^−•^ redox couple is essential for the reaction to take place. However, experiments with *p*-benzoquinone as a superoxide radical scavenger failed, with the opposite effect of enhancing the reaction being observed. It was found that *p*-benzoquinone and oxygen compete for photogenerated electrons in the conduction band of titania. A redox equilibrium between *p*-benzoquinone and hydroquinone (H_2_Q), mediated by the O_2_/O_2_^−•^ system, was identified as a key factor in enabling the reaction. Furthermore, EPR spin-trapping experiments confirmed the presence of the carbon-centered radical 2-hydroxypropan-2-yl, which was determined to be the main radical species involved in the process. Either acetone or 2-propanol can generate this radical, with the BQ/H_2_Q redox system being pivotal in the formation of the hemiacetal intermediate. This intermediate is subsequently converted into the final acetal (solketal), with H_2_Q acting as a photoacid through an excited-state proton transfer (ESPT) mechanism. The photoacid behavior of hydroquinone was confirmed using pyridine as a basic probe, as the formation of hydroquinone–pyridine adducts was detected by Raman spectroscopy.

## 1. Introduction

Current environmental concerns associated with the depletion of fossil fuel resources and the growing threat of climate change have compelled the scientific community and industry to explore sustainable energy alternatives [1]. In this context, biofuels have emerged as a promising solution, with biodiesel gaining particular attention due to its lower carbon footprint and compatibility with existing fuel infrastructures [2]. However, the large-scale production of biodiesel results in the generation of significant amounts of glycerol as by-product. This surplus of glycerol, which often exceeds the demand of its traditional markets, has prompted extensive research into its chemical valorization [3].

**Scheme 1:**
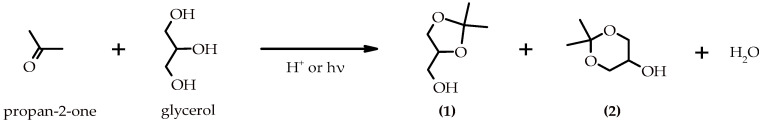
Reaction scheme for the photoacetalization of acetone with glycerol to produce (2,2-dimethyl-1,3-dioxolan-4-yl)methanol (solketal) (**1**) and 2,2-dimethyl-1,3-dioxan-5-ol (DMDO) (**2**).

Glycerol is a highly functionalized and versatile platform molecule that can serve as a precursor to a wide variety of value-added chemicals. Its transformation through selective catalytic processes offers an opportunity to convert a waste stream into commercially useful compounds, contributing to both economic and environmental sustainability [4,5]. Among the various routes explored, the acetalization of glycerol with acetone to produce solketal has emerged as a particularly promising approach (Scheme 1) [6,7].

In this reaction, solketal (the five-membered cyclic acetal) is the predominant product at equilibrium as it is obtained under thermodynamic control. Conversely, the DMDO (the six-membered cyclic acetal) is the kinetically favored product, and its production primarily occurs during the initial stages of the reaction [8]. Solketal exhibits excellent properties as a green solvent and as a fuel additive [9,10]. Regarding fuel applications, solketal has been shown to improve cold flow properties, reduce gum formation, and enhance oxidative stability which are attributes that support the integration of renewable compounds into the energy matrix [11].

Traditionally, the synthesis of solketal has been achieved using acid catalysis, involving either homogeneous or heterogeneous acidic systems [10,12,13,14]. Although effective, these approaches typically require elevated temperatures and may suffer from drawbacks such as catalyst corrosion, separation difficulties, and limited reusability. In recent years, photocatalysis has emerged as a promising alternative to conventional methods, since it operates under milder reaction conditions of ambient temperature and pressure, offering substantial energy savings and reduced environmental impact [15]. The ability to harness light to drive chemical transformations aligns with the principles of green chemistry and has opened new perspectives for the synthesis of solketal and related compounds.

In a previous study conducted by our research group, we demonstrated the feasibility of solketal production via the photoacetalization of acetone with glycerol using commercial titanium dioxide (Aeroxide Evonik P25) as a photocatalyst [15]. The reaction was carried out at room temperature and under ambient air atmosphere (21% O_2_), providing significant yields of solketal. Optimization of the glycerol/acetone molar ratio, along with electron paramagnetic resonance (EPR) spectroscopic analyses, enabled us to propose a reaction mechanism involving the generation of a glycerol-based radical species. This radical was found to attack the carbonyl group of acetone, leading to the formation of an intermediate adduct that later cyclized to form solketal.

Building on these findings, a subsequent study [16] was developed to focus on elucidating the role of oxygen in the photocatalytic process. It was found that the absence of molecular oxygen completely inhibited the reaction, underscoring its essential role in photocatalytic activation. EPR measurements further revealed the formation of superoxide radicals (O_2_^−•^) under irradiation, which were proposed to be key intermediates in the reaction mechanism. To validate the hypothesis that superoxide radicals are crucial to the process, *p*-benzoquinone (BQ) was introduced as a known scavenger of O_2_^−•^ species [17,18,19], with the expectation that it would suppress the formation of solketal by quenching these reactive intermediates.

Interestingly, the addition of *p*-benzoquinone revealed a more complex behavior than initially anticipated. At low concentrations (below 5 mM), *p*-benzoquinone exhibited the expected inhibitory effect, significantly reducing solketal yields and thereby confirming its role as an effective superoxide radical scavenger. However, at higher concentrations, a surprising enhancement in solketal production was observed, suggesting that *p*-benzoquinone may play a more active role in the photoacetalization process beyond simple radical quenching. These intriguing results raise important questions regarding the precise role of *p*-benzoquinone in the photoacetalization of acetone with glycerol. While its inhibitory effect at low concentrations is consistent with the suppression of superoxide-mediated pathways, its ability to promote the reaction at higher concentrations points toward a more nuanced function, possibly involving redox mediation, electron transfer facilitation, or participation in alternative photochemical mechanisms.

Despite the existence of numerous studies on the photochemical behavior of *p*-benzoquinone under different experimental conditions (presence/absence of photocatalyst, oxygen content, presence of organic hydrogen donors, etc.), the majority of these studies have been conducted in aqueous media [19,20,21,22].

There is a general consensus on the behavior of *p*-benzoquinone in aqueous solutions after being irradiated in the absence of a photocatalyst (photolysis) that points to the excitation of benzoquinone to a singlet state ^1^(BQ)* which subsequently transforms into a triplet state ^3^(BQ)* through intersystem crossing: Equation (1). This triplet ^3^(BQ)* state can thereafter revert to the ground state or be transformed/degraded into other organic products [19]. Thus, *Gorner* et al. have reported that in the presence of a hydrogen donor such as propan-2-ol, the triplet ^3^(BQ)* is reduced to hydroquinone (H_2_Q) with a quantum yield close to unity: Equation (2) [23].

(1) BQ→hvBQ*1→ISCBQ*3

(2) BQ*3→iPrOHH2Q

In the presence of a photocatalyst (photocatalysis), benzoquinone is expected to undergo a faster transformation than under photolysis conditions, mainly due to the presence of electron/hole pairs formed in the semiconductor after its excitation. Thus, it has been reported that, in the presence of TiO_2_ P25, *p*-benzoquinone is completely transformed into hydroquinone through photogenerated electrons formed in the conduction band of TiO_2_ after irradiation in the presence of a hydrogen donor, such as propan-2-ol: Equations (3) and (4) [19].

(3) $$TiO_{2}\overset{hv}{\to }TiO_{2}\left(e_{cb}^{-}+h_{vb}^{+}\right)$$

(4) $$BQ\overset{i\mathrm{Pr}OH}{\underset{e_{cb}^{-}}{\to }}H_{2}Q$$

In the context of *p*-benzoquinone’s behavior in relation to its concentration in the reaction medium, *Von Sonntag* et al. have reported that the fate of the ^3^(BQ)* triplet varies depending on the concentrations of BQ in aqueous media [22]. For concentrations below 2 mM, ^3^(BQ)* degrades through reaction with H_2_O or O_2_ molecules, giving rise to polyhydroxy compounds, but without the intervention of other radical species associated to the quinone. Conversely, at elevated concentrations of BQ, the formation of semiquinone radicals SQ^−•^ occurs: Equation (5). Subsequently, in the presence of oxygen, this semiquinone radical can form the superoxide radical (O_2_^−•^) while regenerating the BQ: Equation (6) [18,22,24,25]. Furthermore, in a previous study conducted in an organic medium, the semiquinone radical was detected by EPR spectroscopy [16].

(5) $$BQ\overset{hv}{\to }SQ^{-•}$$

(6) $$SQ^{-•}+O_{2}\to BQ+O_{2}^{-•}$$

*Fonagy* et al. found that working in an aqueous medium and in the absence of a semiconductor, *p*-benzoquinone was transformed into hydroquinone at the same rate regardless of the presence or absence of oxygen (70% transformation of BQ after 4 h of reaction). This observation would indicate that the presence of O_2_ is not necessary for the transformation of BQ into H_2_Q. However, in the presence of a semiconductor (TiO_2_), they found significant differences when the process was carried out in the presence or absence of oxygen. Without O_2_, the transformation of BQ into H_2_Q reached 100% in less than 30 min of reaction and remained constant over the course of the 4 h of experiment. However, in the presence of oxygen, the transformation of BQ into H_2_Q was equally rapid but the concentration of H_2_Q decreased progressively after one hour of reaction as a result of its interaction with superoxide radicals [19].

Furthermore, *Fonagy* et al. reported that when the concentration of *p*-benzoquinone is sufficiently high, it competes with oxygen for the photogenerated electrons in TiO_2_. This competition is effective even if the concentration of BQ (0.25 mM) is comparable to that of dissolved oxygen (0.28 mM at 20 °C). Under these conditions, it is established that the first step in the photocatalyzed process is the scavenging of photogenerated electrons by BQ, which is quantitatively reduced to H_2_Q: Equation (7). However, when the concentration of BQ is below a critical level, dissolved O_2_ begins to be more effective at capturing electrons and forming the corresponding superoxide radical: Equation (8). This superoxide radical could react with BQ to form a semiquinone radical and release O_2_: Equation (9). Semiquinone can undergo additional disproportionation, which in turn yields both BQ and H_2_Q molecules: Equation (10) [19].

(7) $$BQ+2e^{-}+2H^{+}\to H_{2}Q$$

(8) $$O_{2}+e^{-}\to O_{2}^{-•}$$

(9) $$BQ+O_{2}^{-•}\to SQ^{-•}+O_{2}$$

(10) $$2SQ^{-•}+2H^{+}\to BQ+H_{2}Q$$

Once BQ has been reduced, either by electrons (Equation (7)) or by O_2_^−•^ radicals (Equations (8)–(10)), the accumulated H_2_Q starts to degrade, with 1,2,4-trhydroxybenzene being one of the degradation products detected. Furthermore, it has been documented that, in the presence of oxygen, hydroquinone can undergo oxidation by molecular hydrogen, resulting in the formation of benzoquinone, as depicted in Equation (11) [19].

(11) $$H_{2}Q+O_{2}\to BQ+H_{2}O_{2}$$

As illustrated above, it is evident that the behavior of quinones in photocatalytic and photochemical systems is recognized as being multifaceted. Their redox-active nature enables their participation in a variety of electron and proton transfer processes that have the potential to influence the overall catalytic cycle [25,26,27]. However, as previously mentioned, the majority of the reported information has been obtained from experiments conducted in water-based media, with limited data available regarding the behavior of *p*-benzoquinone in organic solvents; therefore, further studies are necessary to elucidate the photochemical behavior of *p*-benzoquinone in them.

The aim of the present work is to gain a deeper understanding of the role of *p*-benzoquinone in the photoacetalization process, carried out in organic media. In particular, the investigation will focus on its dual behavior—as an inhibitor at low concentrations and a promoter at higher ones—by systematically studying its influence in the presence and absence of the Aeroxide Evonik P25 photocatalyst. This study will employ kinetic analysis, spectroscopic characterization, and controlled photocatalytic experiments to explore the mechanistic underpinnings of the reaction and identify the conditions under which *p*-benzoquinone enhances or inhibits solketal formation.

A comprehensive elucidation of these mechanisms is essential not only for optimizing the efficiency of the solketal synthesis but also for advancing the broad field of photocatalytic biomass valorization. Understanding how redox-active additives such as quinones influence photocatalytic pathways could pave the way for the rational design of hybrid catalytic systems that combine photo- and redox-catalysis to achieve higher selectivity, efficiency, and sustainability. Ultimately, this work contributes to the development of innovative strategies for glycerol utilization, aligning with the goals of green chemistry and circular economy.

## 2. Results and Discussion

### 2.1. Influence of the Concentration of p-Benzoquinone

**Figure 1:**
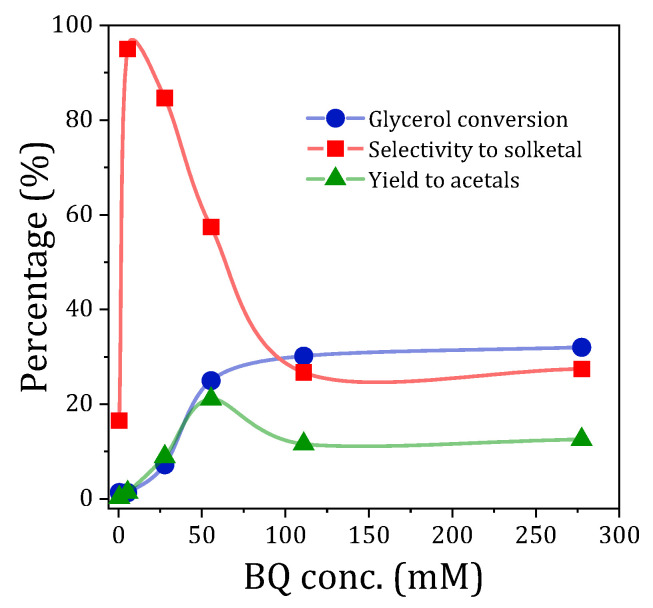
Glycerol conversion, selectivity to solketal, and yield to acetals obtained in the photoacetalization of acetone with glycerol under UV radiation and in the presence of varying amounts of *p*-benzoquinone. Concentration of glycerol, 1 M; reaction time, 2 h.

One of the main questions raised in the use of *p*-benzoquinone (BQ) in the photocatalyzed acetalization reaction of glycerol with acetone to produce solketal is whether BQ acts as a stoichiometric reagent, sensitizer, or as (photo)catalyst. To obtain information in this regard, photoacetalization experiments were carried out with different concentrations of *p*-benzoquinone, in the absence of TiO_2_. The *p*-benzoquinone concentrations that were examined were all clearly substoichiometric (ranging from 0.5 to 278 mM), given that the concentration of glycerol, the limiting reagent, was 1 M in all tests (Figure 1).

The results obtained at a reaction time of 2 h show that, as the BQ concentration increases, the conversion of glycerol increases significantly until reaching a plateau for concentrations above 55.5 mM (30% conversion, 111 mM). However, the solketal selectivity values obtained for the entire range of *p*-benzoquinone concentrations exhibited significant variability, ranging from selectivities approaching 100% for low BQ concentrations to values of 27% for BQ concentrations exceeding 100 mM. As a result of both trends, the yield to acetals shows a maximum (21%) for a *p*-benzoquinone concentration of 55.5 mM. For higher BQ concentrations, a decrease in yield is observed as a result of conversion stagnation and the drop in solketal selectivity. The results are in agreement with those reported in previous studies in which low concentrations of *p*-benzoquinone inhibit the reaction due to their ability to capture superoxide radicals (scavenging effect), while at high concentrations of BQ the observed effect on the photoacetalization reaction is positive, acting as a photocatalyst [16]. However, given that solketal selectivity is compromised at elevated quinone concentrations, 55.5 mM was established as the working concentration for subsequent experiments.

### 2.2. Reaction Profiles with BQ and P25/BQ as Photocatalyst

**Figure 2:**
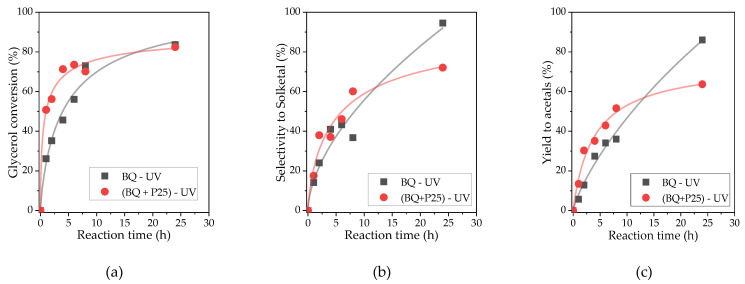
Conversion of glycerol (**a**), selectivity to solketal (**b**), and yield to acetals (**c**) obtained in the photoacetalization of acetone with glycerol under UV light with *p*-benzoquinone or TiO_2_/*p*-benzoquinone as photocatalyst. Reaction conditions: [BQ] = 55.5 mM; [P25] = 2 g/L.

Complete reaction profiles were obtained for the photoacetalization of acetone with glycerol under UV radiation and a *p*-benzoquinone concentration of 55.5 mM, both in the absence and in the presence of TiO_2_ Evonik P25 (Supplementary Figure S1). In both cases, the acetalization reaction reached equilibrium after 24 h, yielding glycerol conversions of 80–85%, consistent with the findings reported in the existing literature [8]. Figure 2 presents a comparative analysis of the glycerol conversions, solketal selectivity, and acetal yield obtained under both reaction conditions.

The profile of the photoacetalization reaction under UV radiation and *p*-benzoquinone as a photocatalyst (Supplementary Figure S1A) demonstrates a continuous decrease in glycerol concentration over time, while the amount of solketal increases almost linearly until reaching values above 85% conversion after 24 h of reaction. Regarding DMDO (six-membered cyclic acetal), it is formed in small quantities during the first 4 h, after which it ceases to be produced and maintains a constant concentration for the rest of the reaction. This behavior is reflected in the selectivity to solketal (Figure 2b), which is low during the initial hours of the reaction due to the high DMDO formation rate (Ssolketal = 8%, 2 h). However, as the reaction progresses, the selectivity increases due to the stagnation in the formation of DMDO (Ssolketal = 95%, 24 h). Regarding the yield of acetals, as the sum of solketal and DMDO, an 85% yield is achieved after 24 h of reaction.

In contrast, when both P25 and *p*-benzoquinone are employed in conjunction as a photocatalytic tandem, the results obtained, though qualitatively similar, exhibit discernible differences in terms of the photoactivity exhibited (Figure 2 and Supplementary Figure S1B). Thus, in the presence of P25/BQ, the rate of glycerol disappearance is high, with a 50% decrease in concentration observed during the initial hour of reaction. Thereafter, the rate slows down, reaching an 85% conversion after 24 h of reaction. The rate of solketal formation is also elevated during the initial 2 h of the reaction, subsequently decelerating during the rest of the process. DMDO displays a comparable behavior to that previously described, forming during the initial two hours of the reaction and then reaching a state of stability at approximately 10%.

It is noteworthy that, following a 24 h reaction period, comparable glycerol conversions are attained for both reaction systems. However, the yields of acetals are notably lower for the P25/BQ system (Yacetal = 64%) in comparison to those obtained for BQ (Yacetal = 85%). This behavior suggests that the presence of TiO_2_ introduces secondary reaction pathways as a result of the interaction of organic compounds with the holes generated on the surface of TiO_2_ under UV irradiation (photodegradation). This degradation has the potential to affect both glycerol and the acetals formed during the process. Furthermore, this degradation may be accentuated at high reaction times as a result of the formation of reactive oxygen species (ROS) from the water formed as a by-product during photoacetalization. In fact, the oxidative degradation of glycerol under UV irradiation in the presence of TiO_2_ has been widely reported and typically involves the formation of oxygenated intermediates such as glyceraldehyde, glycolaldehyde, dihydroxyacetone, formic acid, and acetic acid, eventually leading to CO_2_. These non-selective oxidation pathways are promoted by photogenerated holes and surface-bound ^•^OH radicals, and may compete with the desired photoacetalization process, thus reducing the selectivity of the reaction. A recent study by Herrera-Beurnio et al. demonstrated that glycerol oxidation on TiO_2_ under UV light leads to the accumulation of several of these intermediates [28]. The use of BQ alone prevents such undesirable reactions, ultimately maintaining a more controlled and selective environment for the formation of solketal as the main product.

**Figure 3:**
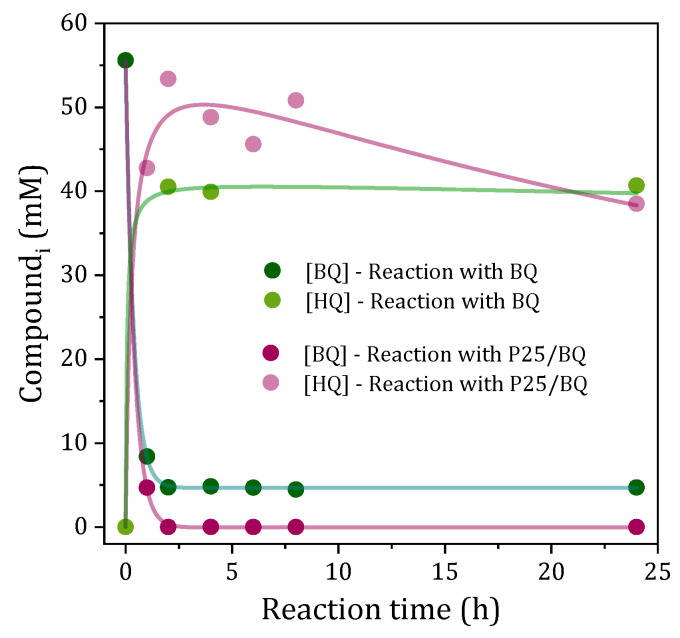
Evolution of concentrations of *p*-benzoquinone and hydroquinone during the photoacetalization of acetone with glycerol under UV light using BQ or P25/BQ tandem.

With the aim of investigating the role of *p*-benzoquinone in the photoacetalization reaction of glycerol with acetone, its evolution was monitored in reactions carried out with BQ or P25/BQ (Figure 3). In the case of the reaction carried out with BQ alone, it was observed that the *p*-benzoquinone content decreased rapidly during the first hour of the reaction until reaching a BQ concentration of around 8% of its initial concentration, which remained constant for the rest of the process. In order to understand the reasons why, under these conditions, *p*-benzoquinone does not fully disappear from the system, it is necessary to consider an equilibrium involving Equations (1), (2) and (11). Moreover, in the case of the reaction carried out with the P25/BQ tandem, the *p*-benzoquinone completely disappeared after the first hour of reaction, according to the results reported in [19]. In both cases, hydroquinone (H_2_Q) is detected as main product of the transformation of *p*-benzoquinone.

However, after 24 h of reaction, the mass balance indicated that the sum of the concentrations of BQ and H_2_Q only attained 82% and 60% of the initial *p*-benzoquinone for the reactions developed with BQ and P25/BQ, respectively. Only low concentrations of 1,2,4-trihydroxybenzene were detected as an additional reaction product, confirming that under the reaction conditions tested, degradation of BQ or H_2_Q also occurs, especially when TiO_2_ P25 was used as a photocatalyst. Again, the presence of water resulting from the formation of the acetal may intensify the photodegradation of BQ/H_2_Q. This observation is consistent with previous reports showing that, under UV irradiation in the presence of TiO_2_, benzoquinone is rapidly converted to hydroquinone and further degraded to higher hydroxylated aromatics such as 1,2,4-trihydroxybenzene, along with unidentified aliphatic and potentially polymeric by-products [29].

These results indicate that a redox equilibrium is established between *p*-benzoquinone and hydroquinone, probably with the participation of the O_2_/O_2_^−•^ system (Equations (1), (2) and (7)–(11)). The presence of TiO_2_ in the reaction media shifts this equilibrium towards hydroquinone and forces the oxidation (photodegradation) processes of the organic species. A particularly noteworthy outcome of these observations relates to the reaction developed with P25/BQ. Despite the complete disappearance of *p*-benzoquinone after the initial hour of reaction, this does not have a substantial impact on the progress of the photoacetalization process that continues until it attains the previously documented acetal yield values after a 24 h reaction period (see Supplementary Figure S2).

### 2.3. Consecutive Addition of p-Benzoquinone and P25

**Figure 4:**
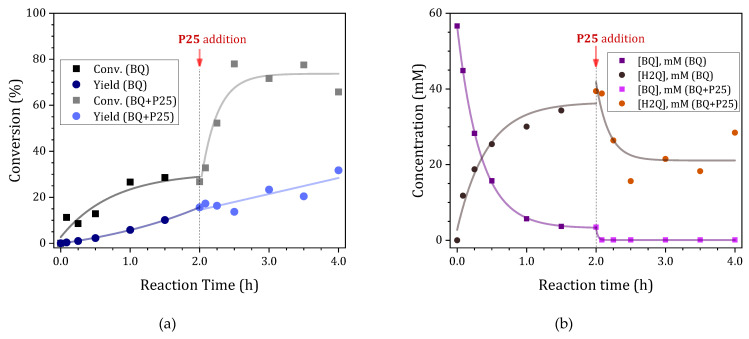
Effect of the addition of TiO_2_ P25 to the reaction medium on the UV-driven photoacetalization of acetone with glycerol using BQ (55.5 mM) as photocatalyst. (**a**) Glycerol conversion and yield to acetals and (**b**) concentration of *p*-benzoquinone and hydroquinone.

In order to corroborate the aforementioned findings, an additional experiment was conducted to analyze the response of the reaction in the presence of *p*-benzoquinone (55.5 mM). The reaction was allowed to proceed for two hours, after which P25 was added to the reaction medium at a concentration of 2 g/L. The results obtained in this experiment are shown in Figure 4 and in Supplementary Figure S3.

Initially, as previously evidenced, the conversion of glycerol and the yield to acetals in the presence of *p*-benzoquinone undergo a gradual increase during the initial two hours of the reaction. Concurrently, the concentration of BQ undergoes a rapid decline due to its conversion to H_2_Q in the presence of UV radiation and a hydrogen donor, such as propan-2-ol (Equations (1) and (2)). In the presence of oxygen, H_2_Q can be re-oxidized to BQ according to Equation (11). This dynamic equilibrium is the underlying reason why a certain amount of *p*-benzoquinone remains detectable during the reaction. Additionally, the mass balance (BQ + H_2_Q) is found to be negative, indicating the formation of intermediate species through a photolytic degradation process (Supplementary Figure S3B). It is plausible that this degradation begins with the formation of the semiquinone radical (SQ^−•^) that can interact with water molecules formed during the acetalization process to yield more hydroxylated species, such as 1,2,4-trihydroxybenzene [19].

At this point, the addition of P25 resulted in a substantial enhancement in glycerol conversion (Figure 4a), accompanied by a notable decline in solketal selectivity (Supplementary Figure S3A). Consequently, the observed decline in selectivity led to a deviation in the correlation between the glycerol conversion and the yield to acetals. This suggests that the addition of P25 induces the oxidation of glycerol to degradation products, presumably due to the action of photogenerated holes on the TiO_2_ surface, thus diverting the reaction to products other than solketal.

Moreover, the addition of TiO_2_ P25 resulted in the complete disappearance of *p*-benzoquinone. This phenomenon, as previously mentioned, can be attributed to the direct reduction of BQ by the photogenerated electrons in P25, thereby disrupting the pre-existing equilibrium between BQ and H_2_Q. Additionally, the negative mass balance for the sum of BQ and H_2_Q may be attributable to the formation of semiquinone and/or the degradation of these species on the catalyst surface. This behavior suggests a change in the dominant reaction mechanism in the presence of P25, favoring alternative oxidation pathways that divert the reaction toward products other than solketal. It is evident that the process of glycerol transformation into solketal persists despite the complete absence of *p*-benzoquinone, again suggesting that the addition of P25 to the reaction medium somehow modifies the mechanism of the photoacetalization reaction.

**Figure 5:**
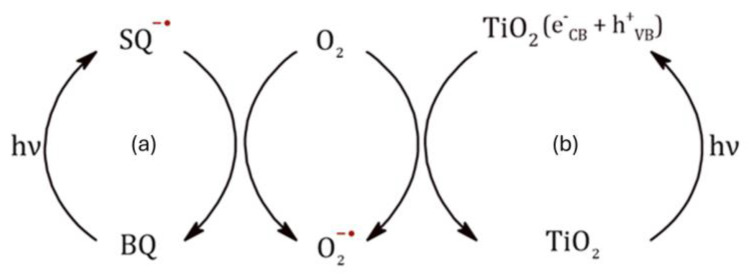
Proposed mechanism of superoxide radical formation after irradiation with UV light in the presence of (**a**) *p*-benzoquinone or (**b**) TiO_2_ as photocatalyst.

Since, whether using BQ or TiO_2_, in the absence of O_2_ the acetalization reaction does not take place and, since the presence of superoxide radicals were detected by EPR in the process [16], it is very likely that these O_2_^−•^ radicals participate in the photoacetalization process in both reaction conditions. In the absence of P25, superoxide radicals can be formed from BQ via semiquinone (Equations (5) and (6)), while in the presence of P25, O_2_^−•^ radicals would be formed by direct reduction of O_2_ with photogenerated electrons from the TiO_2_ conduction layer (Figure 5) [30].

### 2.4. Role of Hydroquinone

**Figure 6:**
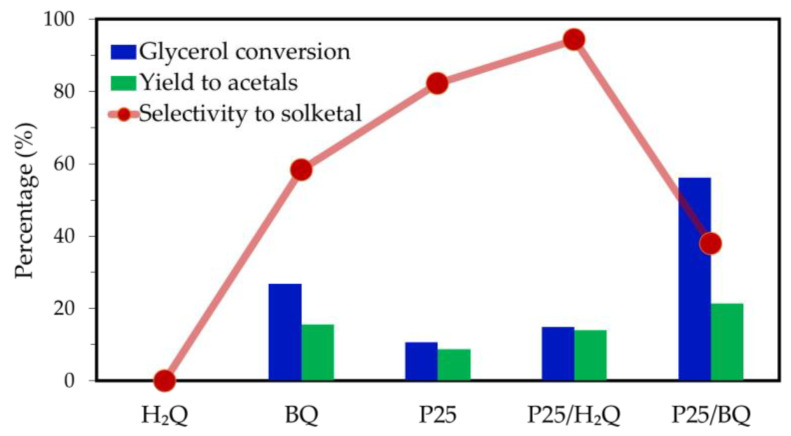
Results obtained in the photoacetalization of acetone with glycerol under UV irradiation when hydroquinone (H_2_Q), *p*-benzoquinone (BQ), TiO_2_ Evonik P25, or a combination of P25/H_2_Q or P25/BQ are used as photocatalyst. Reaction time, 2 h.

At this point, the results confirm that *p*-benzoquinone is a photocatalyst, although under the reaction conditions an equilibrium is established where benzoquinone is mostly converted into hydroquinone. It is also possible that hydroquinone plays an active role in the reaction process, but further investigation is needed. To delve into this hypothesis, the photoacetalization of acetone with glycerol when hydroquinone is used alone or in combination with TiO_2_ (P25/H_2_Q) as photocatalysts was studied. The results obtained are presented in Figure 6, in addition to those obtained using BQ alone, P25, and the P25/BQ tandem, for comparison.

Firstly, after 2 h of reaction in the presence of hydroquinone, no acetalization reaction products were detected. However, when hydroquinone is used in combination with P25, an acetal yield of 14% is obtained, which is higher than that obtained with P25 alone (Yacetal = 8%). In addition, the results obtained with the P25/H_2_Q tandem are equivalent to those obtained with BQ alone (Yacetal = 15%), although both are somewhat lower than those obtained by the P25/BQ combination (Yacetal = 21%). The enhancement in activity observed when H_2_Q is used with TiO_2_ reinforces the hypothesis that hydroquinone plays an active role in the process, at least when used in combination with TiO_2_.

Regarding solketal selectivity, the P25/H_2_Q combination demonstrates high selectivity (Ssolketal, 94%), while reactions occurring in the presence of P25/BQ exhibit significantly lower selectivity (Ssolketal, 38%). Consequently, while the P25/BQ combination converts glycerol partly to reaction products other than solketal, the P25/H_2_Q tandem minimizes the conversion of glycerol to by-products. The behavior of the P25/BQ system can be explained by considering the interaction of BQ with the photogenerated electrons in TiO_2_, in such a way that BQ acts as a redox mediator that facilitates the closure of the oxidation–reduction cycle through its reduction to H_2_Q (Equation (7)). Concurrently, the holes photogenerated in TiO_2_ are primarily utilized to oxidize glycerol to by-products, thereby completing the cycle. This process increases glycerol conversion but diverts the reaction towards oxidation pathways that do not favor solketal formation. As a result, there is a decrease in solketal selectivity and acetal yield.

**Figure 7:**
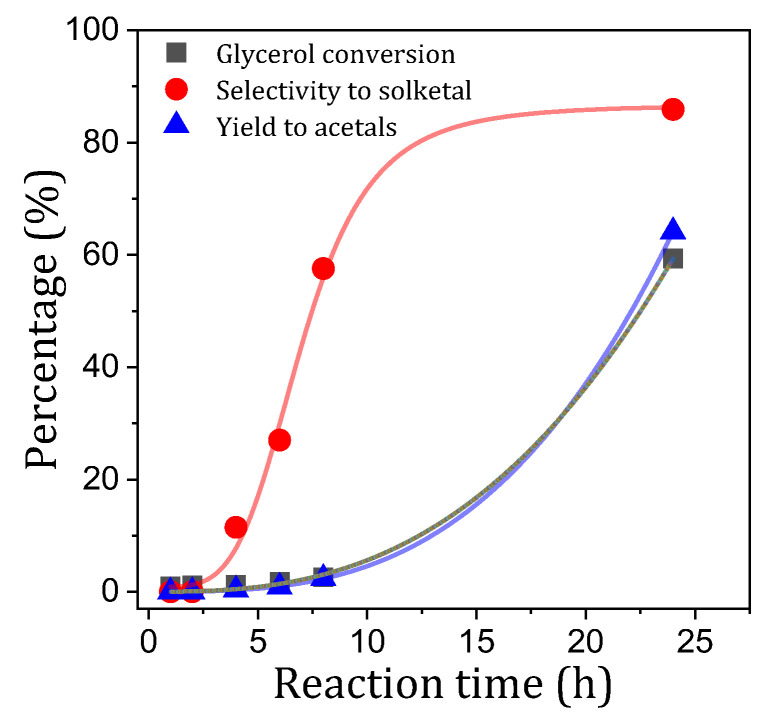
Photoacetalization of acetone with glycerol using hydroquinone as photocatalyst.

It is intriguing that, considering the outcomes observed with the P25/H_2_Q combination, hydroquinone alone appears to be ineffective in photoacetalization following a 2 h reaction period. Consequently, a reaction with hydroquinone was conducted at 24 h, the results of which were highly revealing (Figure 7). This profile confirms that up to four hours of reaction, the observed activity is practically nil. However, after 6–8 h of reaction, acetals begin to be detected, so that after 24 h of reaction, the yield to acetals is 64%, with a selectivity to solketal of 86%. The hypothesis proposed for this delay in the onset of the reaction is that when there is 100% hydroquinone, the superoxide radical cannot be formed according to the scheme presented in Figure 5. However, as reaction time increases, it is possible that hydroquinone will gradually form *p*-benzoquinone, ultimately reaching an equilibrium (Equation (11)). Subsequent analysis of samples collected at elevated reaction times revealed *p*-benzoquinone concentrations in the range of 5 mM, corresponding to 8% molar of the initial hydroquinone. This BQ level is analogous to the residual one obtained when BQ was added alone, although in that case, equilibrium was reached more rapidly (approximately after 2 h of reaction). In this scenario, the formed *p*-benzoquinone would initiate the acetalization process.

Conversely, when the reaction is carried out with the P25/H_2_Q system, it is TiO_2_ that is responsible for initiating the acetalization process, and the presence of BQ is not necessary for the process to proceed. However, the role of hydroquinone itself in this process remains to be elucidated, as its use in combination with P25 has been shown to enhance the yield of acetals obtained.

It has been reported that organic molecules with activated phenolic groups, like Eosin Y, are often used in a variety of photocatalytic processes. Yi et al. reported using Eosin Y as a photocatalyst in the light-driven acetalization reaction of aldehydes. However, they did not explore the reaction mechanism [31]. Yan et al. later suggested two new uses for Eosin Y in photochemical reactions. First, it can act as a photoacid through an excited-state proton transfer (ESPT) process. Second, it can activate C-H bonds through a process called hydrogen atom transfer (HAT) [32].

In terms of photoacidity, the hydroquinone molecule contains two hydroxyl groups in positions 1 and 4, which give it amphoteric properties, allowing it to act as both a weak acid and a weak base. Alcohols are classified as weak acids with pKa values ranging from 15 to 18. In contrast, phenols possess higher acidity, with pKa values typically around 10. This higher acidity is justified by the stability that the phenoxide anion achieves through resonance, favoring a certain degree of deprotonation. However, it should be noted that hydroquinone would not be a sufficiently strong acid to activate a carbonyl group to the extent of promoting acetalization.

According to the literature, phenolic compounds have been observed to exhibit photoacid behavior [27,33,34,35,36]. A photoacid is a molecule that, after absorbing a photon of light, experiences a significant increase in acidity, enhancing its ability to transfer a proton from the excited state to an acceptor molecule. In general, the excitation of a photoacid can reduce its pKa by 6–8 units, thereby increasing its acidity by a factor of 10^6^–10^8^. This phenomenon serves as a crucial link between the absorption of a photon and the subsequent release of a proton [33,35,37,38]. For example, the photocatalytic protonation of a silylenol ether using 7-bromo-2-naphthol as an excited-state proton transfer (ESPT) catalyst has been demonstrated [39]. To reach the excited state from which the proton is transferred, the phenol itself can absorb a photon, or alternatively, interact with a photosensitizer by means of an energy transfer process.

To confirm that hydroquinone behaves as a photoacid under the reaction conditions employed, the acid–base interaction of pyridine (a Lewis base) with hydroquinone both in the dark and under UV radiation was studied by Raman spectroscopy. The pyridine band monitored was the skeletal vibration (νs, ν1, A1, symmetric ring breathing) that appears at 991 cm^−1^ for liquid pyridine [40]. When the unshared electron pair of pyridine nitrogen interacts with an acid compound, the position of this band shifts to higher wave numbers. The greater the acid–base interaction recorded, the more pronounced this shift is. Therefore, an interaction via hydrogen bonds (weak) causes a shift of the ν1 symmetric ring breathing band from 991 cm^−1^ to about 996–1008 cm^−1^, depending on the strength of the hydrogen bonds formed. Conversely, if a proton is completely transferred from a Brønsted acid, the ν1 band of the pyridinium cation formed shifts to approximately 1007–1015 cm^−1^. An interaction with a Lewis acid would result in a shift of the signal to 1018–1028 cm^−1^.

**Figure 8:**
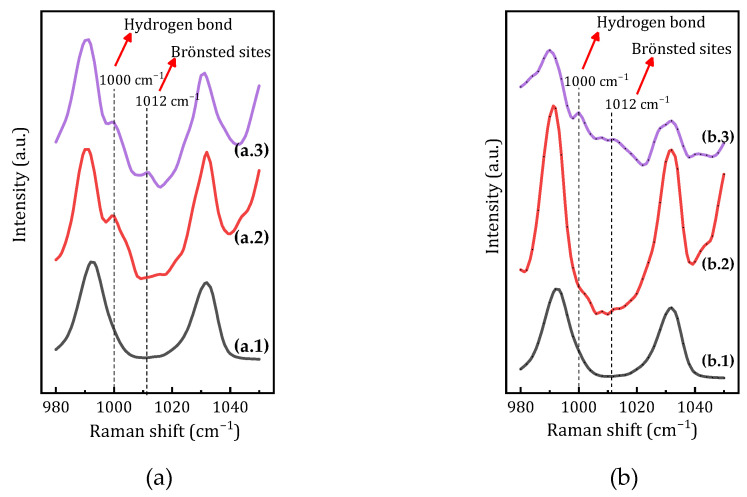
Raman spectroscopy detection of the acid–base interaction of pyridine with (**a**) hydroquinone and (**b**) *p*-benzoquinone. The spectra corresponding to pyridine dissolved in acetone (a.1 and b.1), pyridine plus H_2_Q or BQ in the dark (spectra a.2 and b.2, respectively), and pyridine plus H_2_Q or BQ after 30 min of UV irradiation (spectra a.3 and b.3., respectively) are shown.

In consideration of the above-mentioned factors, a comparative analysis was conducted between the Raman spectrum of an acetone solution of pyridine and the spectra of acetone solutions of pyridine in mixtures with *p*-benzoquinone or hydroquinone. The samples were prepared in a dark environment to obtain the initial Raman spectrum. They were then irradiated with UV light, and the Raman spectra of the mixtures were recorded after 5, 20, and 30 min of irradiation. The results obtained are shown in Figure 8. The pyridine solution in acetone displays the symmetric ring breathing band at 991 cm^−1^, consistent with the characteristic signal of pure pyridine, indicating an absence of acid–base interactions.

When the experiment is carried out with pyridine and hydroquinone, an unambiguous signal at 1000 cm^−1^ is observed immediately after mixing the two substances and while still in the dark. This signal is associated with the hydrogen bonds formed between the pyridine and an -OH group of the hydroquinone. Following the irradiation of the mixture with UV light for 30 min, in addition to the signal at 1000 cm^−1^, a distinct signal at 1012 cm^−1^ is evident. This is attributed to the pyridinium cation, which is formed through the transfer of a proton from the hydroquinone in an excited state, acting as a photoacid (Supplementary Figure S4).

Conversely, when the experiment is conducted with a solution of pyridine and *p*-benzoquinone, the Raman spectrum obtained under dark conditions exhibits no signals above 1000 cm^−1^. However, when the sample is exposed to UV radiation for 30 min, two signals emerge: one at 1000 cm^−1^ associated with the interaction of pyridine via hydrogen bonds, and a second signal at 1012 cm^−1^ associated with the pyridinium cation formed after proton transfer. These bands are associated with the presence of hydroquinone formed during the UV irradiation period of BQ. Again, the signal at 1012 cm^−1^ suggests the transfer of a proton from the excited state of hydroquinone formed during UV irradiation, thereby functioning as a photoacid.

**Figure 9:**
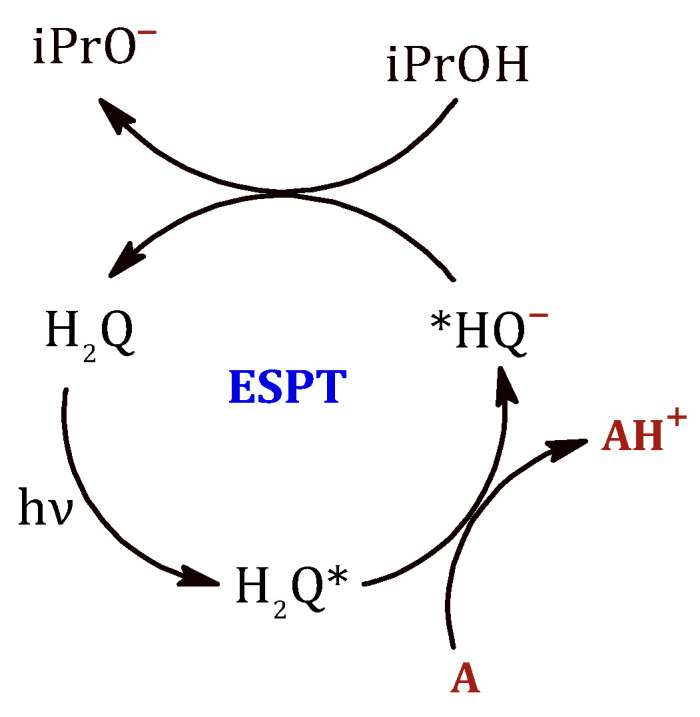
Photoacid behavior of hydroquinone after absorbing a photon of UV radiation. After transfer of the proton to an acceptor molecule (A), hydroquinone is regenerated with the intervention of propan-2-ol present in the reaction medium.

The results demonstrate that when a solution containing hydroquinone is exposed to UV light, it undergoes excitation, resulting in its behavior as a photoacid by transferring a proton to an acceptor molecule (Figure 9). Subsequently, the hydroquinone molecule would be regenerated by the action of the isopropanol present in the reaction medium.

### 2.5. EPR Spin-Trapping Experiments

**Figure 10:**
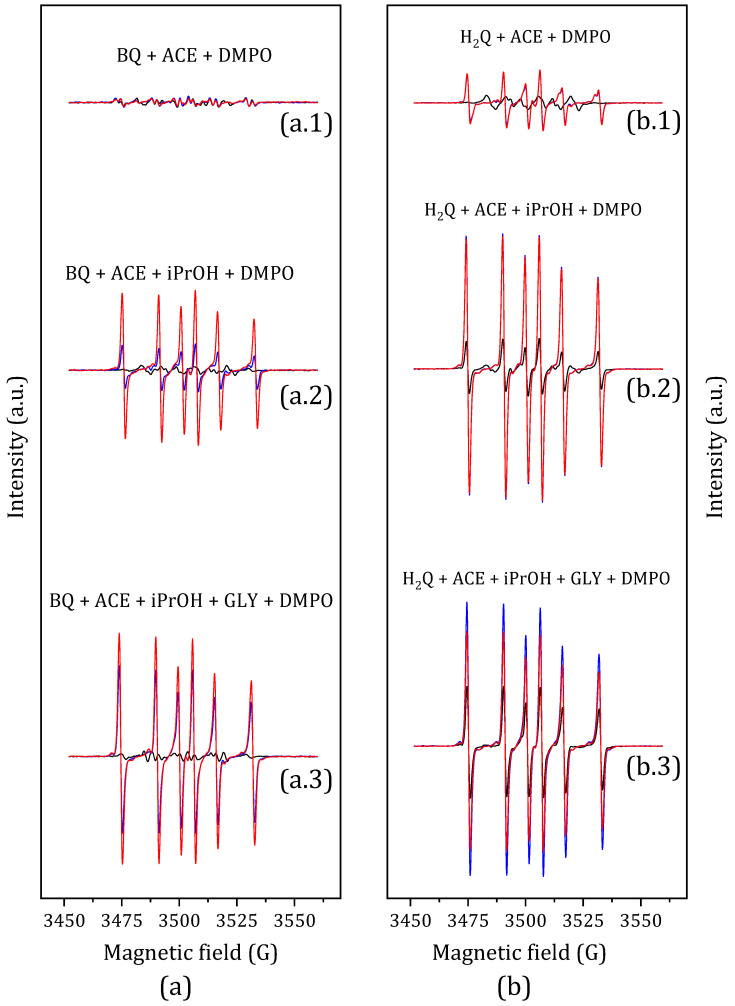
EPR spin-trapping experiments with DMPO of (**a**) *p*-Benzoquinone solutions: (**a.1**) BQ in acetone, (**a.2**) BQ in acetone and propan-2-ol, and (**a.3**) BQ in acetone, propan-2-ol, and glycerol; (**b**) Hydroquinone solutions: (**b.1**) H_2_Q in acetone, (**b.2**) H_2_Q in acetone and propan-2-ol, and (**b.3**) H_2_Q in acetone, propan-2-ol, and glycerol. The samples were exposed to UV light for one minute (black line), 10 min (blue line), and 15 min (red line). All spectra are on the same scale.

To gain deeper understanding of the role of benzoquinone and hydroquinone in the photoacetalization process of acetone with glycerol, a study of the radical species formed during the process was conducted using electron paramagnetic resonance spectroscopy (EPR). This study employed 5,5-dimethyl-1-pyrroline N-oxide (DMPO) as a spin-trapping reagent [41,42]. The results obtained in this study are shown in Figure 10.

The *p*-benzoquinone solution in acetone shows no signal after being irradiated for 15 min with UV light, indicating that no relevant radical species are formed (Figure 10, trace a.1). When the spectrum is obtained in the presence of BQ and propan-2-ol, a signal consisting of a hexaplet clearly develops (Figure 10(a.2)). This signal is also observed when the spectrum is obtained under reaction conditions, including BQ, propan-2-ol, and glycerol (Figure 10(a.3)). Conversely, irradiation of the hydroquinone solution in acetone reveals comparable signals after 15 min, though with reduced intensity (Figure 10(b.1)). These signals are noticeably intensified when propan-2-ol is present in the solution (Figure 10(b.2)), as well as when the spectrum is obtained in the presence of H_2_Q, propan-2-ol, and glycerol (Figure 10(b.3)).

**Figure 11:**
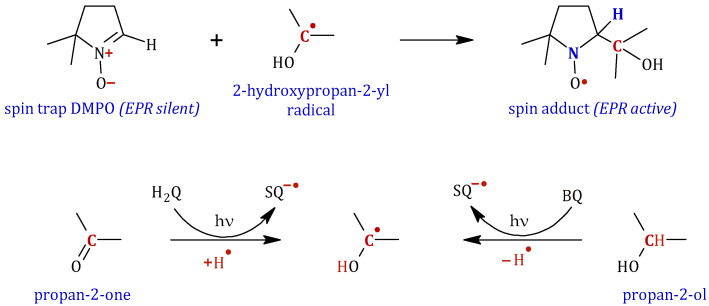
Detail of the formation of the adduct of 2-hydroxypropan-2-yl radical with the spin trap DMPO. Proposed mechanism for the formation of this radical from acetone, with the intervention of hydroquinone, or from propan-2-ol with benzoquinone under UV irradiation.

With regard to the signals detected in all experiments, they are associated with a carbon-centered radical species that supports at least one hydroxyl group, as indicated by its hyperfine coupling constants A_N_ (14.68 G) and A_H_ (23.67 G) [42,43]. Since this radical is detected in the absence of glycerol (spectra a.2 and b.2), it can be deduced that the radical species is not associated with this reagent. Conversely, the absence of detection in the presence of BQ and acetone suggests that, in principle, the radical species is not associated with acetone. Finally, since the presence of propan-2-ol invariably results in the formation of the adduct between the radical and DMPO, all evidence points to the carbon-centered radical being 2-hydroxypropan-2-yl (Figure 11). The hyperfine coupling constants obtained in this experiment (A_N_ 14.68 G; A_H_ 23.67 G; solvent acetone) are reasonably consistent with those found in the literature for the trapping of this radical with DMPO (A_N_ 14.58 G; A_H_ 23.91 G; solvent benzene) (Supplementary Figure S5) [42,44,45].

The formation of the 2-hydroxypropan-2-yl radical can be explained by a hydrogen atom abstraction from propan-2-ol by excited *p*-benzoquinone following a direct hydrogen atom transfer process (HAT), as illustrated in Figure 11 [46,47]. The hydrogen atom transfer (HAT) process is a key step involved in various chemical, environmental, and biological processes [48,49]. As reported by Manfrotto et al., the triplet state anthraquinone has the ability to abstract hydrogen atoms with high efficiency from alcohols, acetals, and even alkanes, thereby generating carbon-centered radicals [50].

Conversely, as noted above, when the H_2_Q solution in acetone is exposed to 15 min of irradiation, analogous signals are detected, though at reduced concentrations (trace b.1). The presence of this radical in the absence of propan-2-ol can be explained by considering a hydrogen atom transfer process from hydroquinone to an acetone molecule, thus forming the EPR-detected radical in spectrum b.1 (Figure 11).

### 2.6. Reaction Mechanism

In light of the results discussed above, a reaction mechanism can be proposed for the photoacetalization of acetone with glycerol in the presence of *p*-benzoquinone or hydroquinone. According to the findings of previous studies [16], the presence of oxygen is necessary for the reaction to occur, thus suggesting the involvement of the superoxide radical in the process. In addition, EPR has provided evidence of the formation of the 2-hydroxypropane-2-yl radical, which can be formed from both acetone and propane-2-ol. Finally, it has been proven that, under reaction conditions, hydroquinone behaves as a photoacid, capable of transferring a H⁺ to an acceptor molecule, while benzoquinone can participate in direct hydrogen transfer (HAT) processes, responsible for the activation of C-H bonds and the formation of carbon-centered radical species.

**Figure 12:**
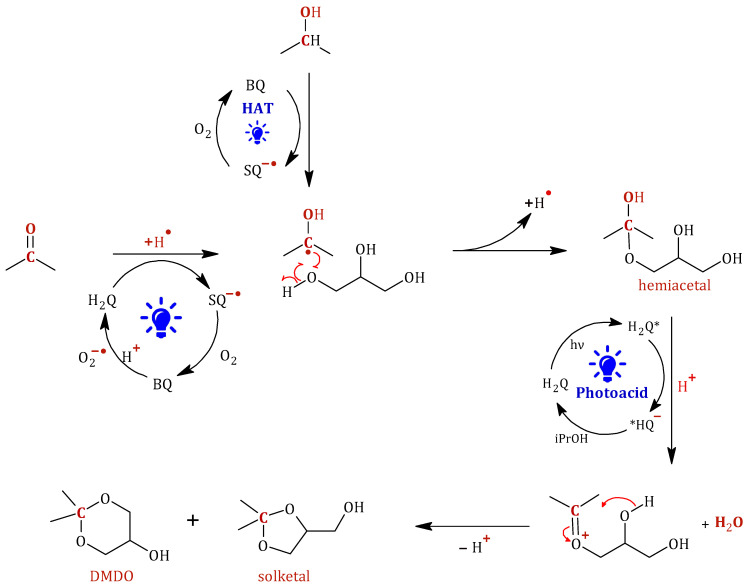
Proposed reaction mechanism for the photoacetalization of acetone with glycerol using *p*-benzoquinone as photocatalyst and under UV irradiation.

Figure 12 presents the proposed reaction mechanism, which reflects the considerations outlined above. The formation of the 2-hydroxypropane-2-yl radical from acetone with the intervention of hydroquinone is proposed. The participation of the O_2_/O_2_^−•^ system at this point is crucial, as it allows the catalytic cycle of the H_2_Q/SQ^−•^/BQ system to be completed. Furthermore, the 2-hydroxypropane-2-yl radical could also be formed from propan-2-ol through an HAT process involving *p*-benzoquinone. Once this radical is formed, it attacks a terminal -OH group of glycerol, resulting in the formation of the corresponding hemiacetal. Following the formation of the hemiacetal, the photoinduced acidity of the hydroquinone would intervene in the completion of the process by protonating the hemiacetalic -OH through an excited-state proton transfer (ESPT) process and thus promoting its dehydration. This would form a positively charged reaction intermediate that would undergo nucleophilic attack by one of the two remaining -OH groups of glycerol, leading to the formation of cyclic acetals (solketal or DMDO). Therefore, the *p*-benzoquinone/semiquinone/hydroquinone system functions as a redox cycle, catalyzing the photoacetalization of acetone with glycerol to yield solketal.

## 3. Materials and Methods

### 3.1. Chemicals

Acetone ≥ 99.5% (Art. 32201M), glycerol ≥ 99.5% (Art. G7893), propan-2-ol ≥ 99.7% (Art. W292907), *p*-benzoquinone 99% (Art. 802410), hydroquinone 99% (Art. H9003), pyridine ≥ 99% (Art. P3776), and 5,5-Dimethyl-1-pyrroline N-oxide (DMPO) 98% (Art. 92688) were procured from Merck and used without any further treatment. Titanium dioxide Aeroxide^®^ TiO_2_ P25 was obtained from Evonik.

### 3.2. Photoacetalization of Acetone with Glycerol

The reaction mixture was composed of a molar ratio of 1:11:2 of glycerol, acetone, and propan-2-ol, used as solvent to homogenize the reaction medium. Photocatalytic experiments were carried out in air atmosphere (21% O_2_) with the required concentration of *p*-benzoquinone in the range of 0.5 to 275 mM (glycerol to *p*-benzoquinone molar ratio from 1729 to 3.5). For the TiO_2_ photocatalyzed reactions, a 2 g/L concentration of Aeroxide^®^ P25 was used.

The samples were analyzed using gas chromatography with N,N-dimethylformamide (DMF) as external standard. An aliquot of 100 μL of sample was extracted, followed by the addition of 19 μL of DMF. The mixture was then filtered using 0.45 μm PTFE filters. The analysis was performed using an Agilent GCMS 5977 Turbo System (Agilent, Santa Clara, CA, USA) equipped with a 30 m HP-INNOWax capillary column and quadrupole mass selective detector (MSD). Calibration curves for glycerol and solketal were obtained using N,N-dimethylformamide as an external standard. The glycerol conversion selectivity to solketal and acetals yield were calculated using the following equations:

(12) $$Glycerol\;conversion\left(\%\right)=\frac{moles\;of\;glycerol\;converted}{initial\;mole\;of\;glycerol}\times 100$$

(13) $$Selectivity\;to\;solketal\left(\%\right)=\frac{moles\;of\;solketal\;formed}{moles\;of\;glycerol\;converted}\times 100$$

(14) $$Yield\;to\;acetals\left(\%\right)=\frac{moles\;of\;solketal+moles\;of\;DMDO\;formed}{initial\;mole\;of\;glycerol}\times 100$$

The liquid-phase batch photoacetalization of acetone with glycerol was conducted in a 3-mouth heart-shaped photoreactor with a total volume of 25 mL and a reaction volume of 10 mL. Reactions were carried out at room temperature under UV light (UV Spotlight Source Lightningcure™ L8022, Hamamatsu, Hamamatsu, Japan), with the light focused on the sample compartment through an optical fiber. The UV lamp had a maximum emission of 365 nm, with a radiant flux of 0.25 W.cm^−2^ in the reaction compartment, as measured using a UV-meter Model 818P-015-19 (Newport, Irvine, CA, USA). UV spectral distribution of the UV lamp used can be found in Supplementary Figure S6.

### 3.3. EPR Trapping Measurements

The radicals generated during the photocatalytic reaction were analyzed using electron paramagnetic resonance (EPR) spectroscopy, employing 5,5-dimethyl-1-pyrroline N-oxide (DMPO) as a trapping agent. The samples were analyzed using a Bruker EMX-Micro X-band EPR spectrometer (Bruker Española S.A., Madrid, Spain) operating at a frequency of 9.81 GHz. The modulation parameters were set at 100 kHz for the field frequency, 1 G for the modulation amplitude, and 0.5 mW for the microwave power.

The spectra were recorded in air at ambient temperature using a micropipette with an internal diameter of 3 mm (Blaubrand^®^ intraMARK, Sigma-Aldrich, St. Louis, MO, USA). The samples analyzed consisted of a 0.55 M solution of either BQ or H_2_Q in acetone, acetone/propan-2-ol (11:2 molar ratio) or acetone/propan-2-ol/glycerol (1:11:2 molar ratio), to which DMPO was added at a concentration of 0.018 M. EPR spectra were recorded after exposing the sample to irradiation with a UV lamp at intervals of 0, 5, 10, 15, and 20 min.

### 3.4. Raman Spectroscopic Studies of Acid–Base Interaction of H_2_Q with Pyridine

The study of the acid–base interaction between pyridine (Lewis base) and hydroquinone as photoacid was conducted using Raman spectroscopy. Raman spectra were obtained using an EZ-Raman spectrometer (En-Wave Optronics Inc., Irvine, CA, USA) equipped with a 785 nm laser focused with a high-throughput fiber optics probe. Solutions of pyridine (0.12 M) in acetone were prepared in a dark environment, followed by the addition of hydroquinone (0.5 M) or benzoquinone (9 mM) as needed. The solutions, placed in a 2 mL vial, were exposed to UV light for 0, 5, 20, and 30 min, and the Raman spectrum was obtained after each irradiation period.

## 4. Conclusions

This work has focused on the photocatalyzed synthesis of solketal under UV radiation from a mechanistic perspective. This study utilized *p*-benzoquinone or TiO_2_ P25/*p*-benzoquinone as photocatalysts. It has been established that during the reaction, when *p*-benzoquinone is used alone, a redox equilibrium is established between *p*-benzoquinone and hydroquinone with the participation of the O_2_/O_2_^−•^ system. This equilibrium is crucial for understanding the evolution of the photoacetalization process. The presence of TiO_2_ in the reaction shifts this equilibrium towards hydroquinone and forces the oxidation (photodegradation) processes of the organic species. EPR spin-trapping experiments with DMPO have revealed that the main radical species obtained is 2-hydroxypropan-2-yl radical, which would initiate the process leading to the formation of the corresponding hemiacetal. This radical could be formed from acetone with the participation of hydroquinone, as well as from propan-2-ol with the participation of *p*-benzoquinone. It has been proven that hydroquinone, under reaction conditions (UV radiation), can develop excited-state proton transfer (ESPT) processes to an acceptor species, thus behaving as a photoacid. This behavior has been verified using pyridine as a basic probe molecule, detecting the hydroquinone/pyridine adducts by Raman spectroscopy. It has been proposed that, under reaction conditions, photoacid hydroquinone protonates the hemiacetal OH, thereby promoting its dehydration and facilitating the transformation of the hemiacetal into acetal (solketal and DMDO).

## Data Availability

The data used to support the findings of this study can be made available by the corresponding author upon request.

## Supplementary Materials

The following supporting information can be downloaded at: https://www.mdpi.com/article/10.3390/molecules30163339/s1, Supplementary Figure S1. Reaction profiles obtained in the photoacetalization of acetone with glycerol under UV-light with (A) *p*-benzoquinone and (B) TiO_2_/*p*-benzoquinone as photocatalysts. Supplementary Figure S2. Comparison between the evolution of *p*-benzoquinone concentration and yield to acetals obtained in the photoacetalization of acetone with glycerol under UV radiation on BQ (A) or P25/BQ (B). Supplementary Figure S3. Effect of the addition of TiO_2_ P25 to the reaction medium on the selectivity to solketal (A) and the *p*-benzoquinone plus hydroquinone mass balance (B) during the UV-driven photoacetalization of acetone with glycerol using *p*-benzoquinone (55.5 mM) as photocatalyst. Supplementary Figure S4. Interactions of pyridine as a Lewis base with H_2_Q-related species: hydrogen bonding with the -OH group of hydroquinone (top), or proton transfer from a hydroquinone molecule acting as a photoacid (bottom). The Raman shift at which the pyridine symmetric ring breathing band (ν1) appears is shown for each species involved. Supplementary Figure S5. Calculation of hyperfine coupling constants (AN and AH) in EPR spin-trapping experiments with DMPO. Supplementary Figure S6. UV spectral distribution of UV spotlight from Hamamatsu (365 nm type blue trace, type [-01A]) as provided by the supplier (Hamamatsu, Shizuoka Pref., Japan).

## Abbreviations

The following abbreviations are used in this manuscript:

BQ	*p*-Benzoquinone
H_2_Q	Hydroquinone
Solketal	(2,2-dimethyl-1,3-dioxolan-4-yl)methanol
DMDO	2,2-dimethyl-1,3-dioxan-5-ol
DMF	N,N-dimethylformamide
DMPO	5,5-dimethyl-1-pyrroline N-oxide
iPrOH	Isopropanol; propan-2-ol
GLY	Glycerol
ACE	Acetone; propan-2-one
ESPT	Excited-state proton transfer
HAT	Hydrogen atom transfer
