# Supplementary material for: Role of p-Benzoquinone in the Photocatalytic Production of Solketal
Source: Molecules. 2025 Aug 11;30(16):3339. doi: 10.3390/molecules30163339 (PMC12388013; doi:10.3390/molecules30163339)
Supplement: Supplementary file 1 [file molecules-30-03339-s001.zip › molecules-3799599-supplementary.pdf]

# Role of p-benzoquinone in the photocatalytic production of solketal

**Juan Martín-Gómez[1], Alejandro Ariza-Pérez[1], M. Carmen Herrera-Beurnio[1], Francisco J. López-Tenllado[1], Jesús Hidalgo-Carrillo[1], Alberto Marinas[1] and Francisco J. Urbano [1*]**

[1] Departamento de Química Orgánica, Instituto Químico para la Energía y el Medioambiente (IQUEMA), Universidad de Córdoba, E-14071 Córdoba, Spain
* Correspondence: FJU, FJ.Urbano@uco.es

## Index

S1. Reaction profiles in photoacetalization of acetone with glycerol .................................................. 2
S2. Evolution of BQ concentration and yield to acetals ........................................................................ 3
S3. Effect of $TiO_2$ P25 addition after 2h of reaction in the presence of BQ ......................................... 4
S4. Interactions of pyridine (Lewis base) with excited hydroquinone (photoacid) ........................... 5
S5. Hyperfine coupling constants ($A_N$ and $A_H$) in EPR spin trapping experiments with DMPO..... 6
S6. Details of the UV lamp used in the process ..................................................................................... 7

## S1. Reaction profiles in photoacetalization of acetone with glycerol

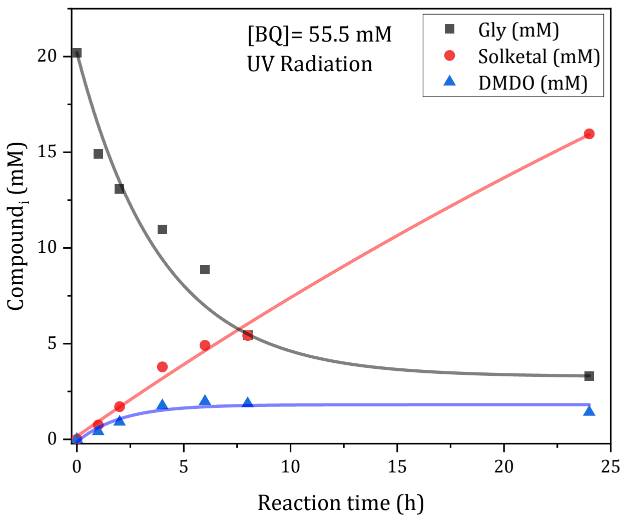


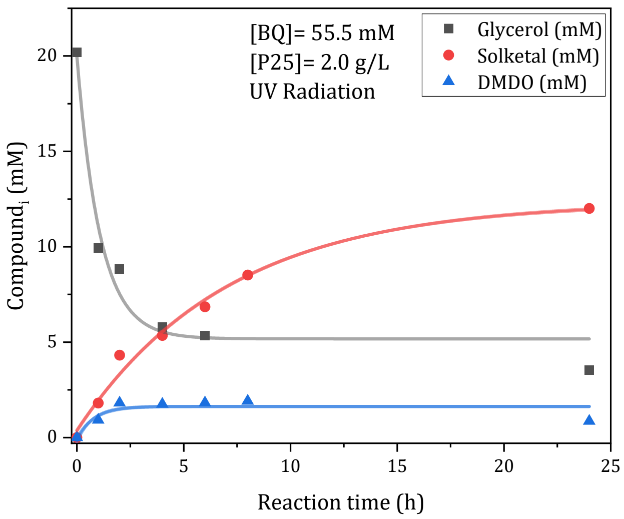


Supplementary Figure S1. Reaction profiles obtained in the photoacetalization of glycerol with acetone under UV-light with p-benzoquinone (A) and $TiO_2$/*p*-benzoquinone (B) as photocatalyst and or photosensitiser.

## S2. Evolution of BQ concentration and yield to acetals

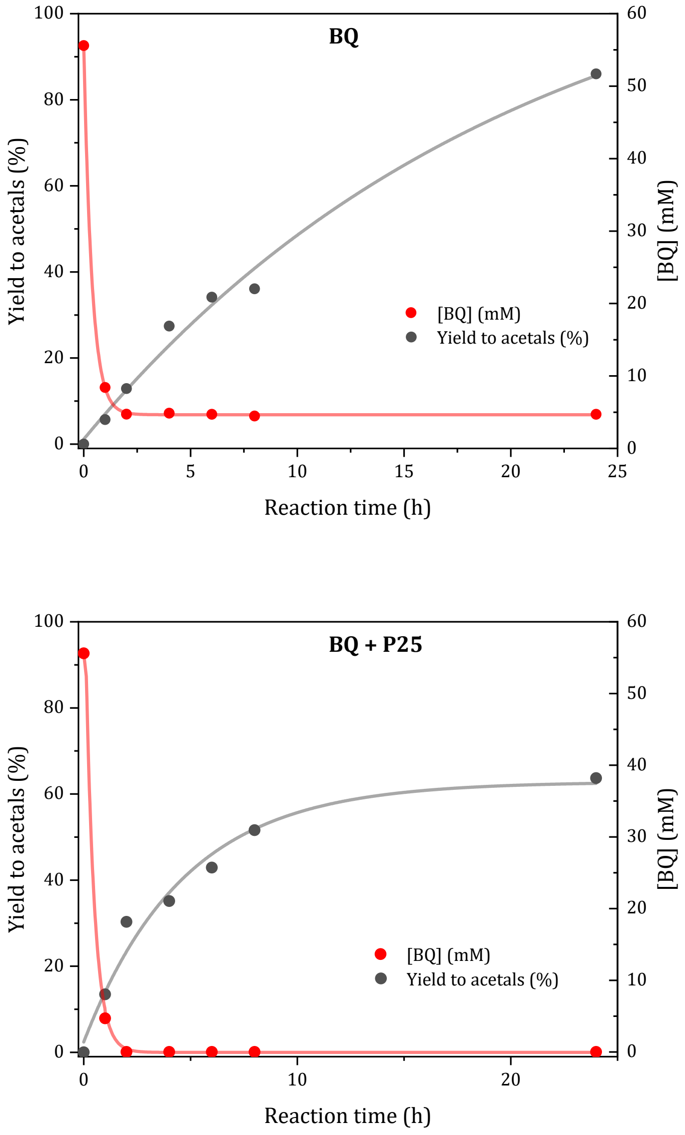


Supplementary Figure S2. Comparison between the evolution of *p*-benzoquinone concentration and yield to acetals obtained in the photoacetalization of glycerol with acetone under UV radiation on BQ (A) or P25/BQ (B).

## S3. Effect of $TiO_2$ P25 addition after 2h of reaction in the presence of BQ

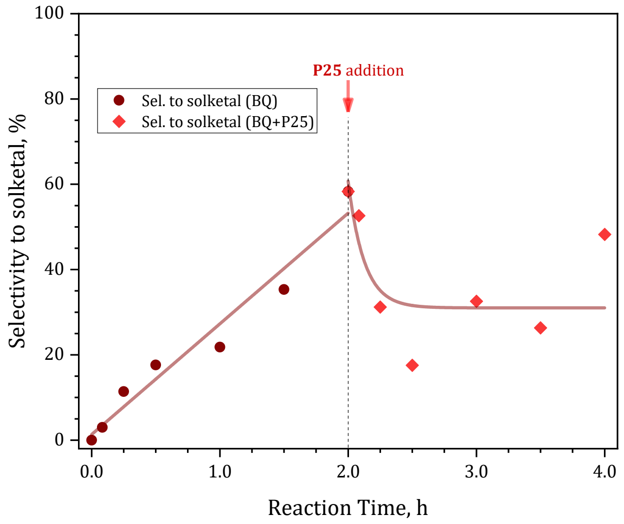


v

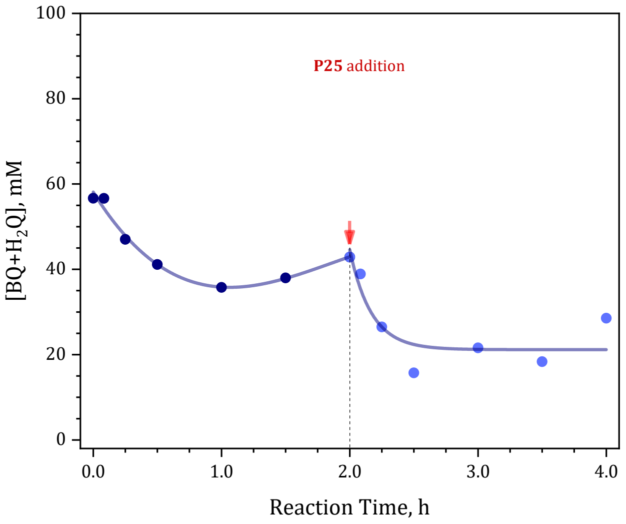


Supplementary Figure S3. Effect of the addition of $TiO_2$ P25 to the reaction medium on the selectivity to solketal (A) and the *p*-benzoquinone plus hydroquinone mass balance (B) during the UV-driven photoacetalization of acetone with glycerol using *p*-benzoquinona (55.5 mM) as photosensitiser.

## S4. Interactions of pyridine (Lewis base) with excited hydroquinone (photoacid)

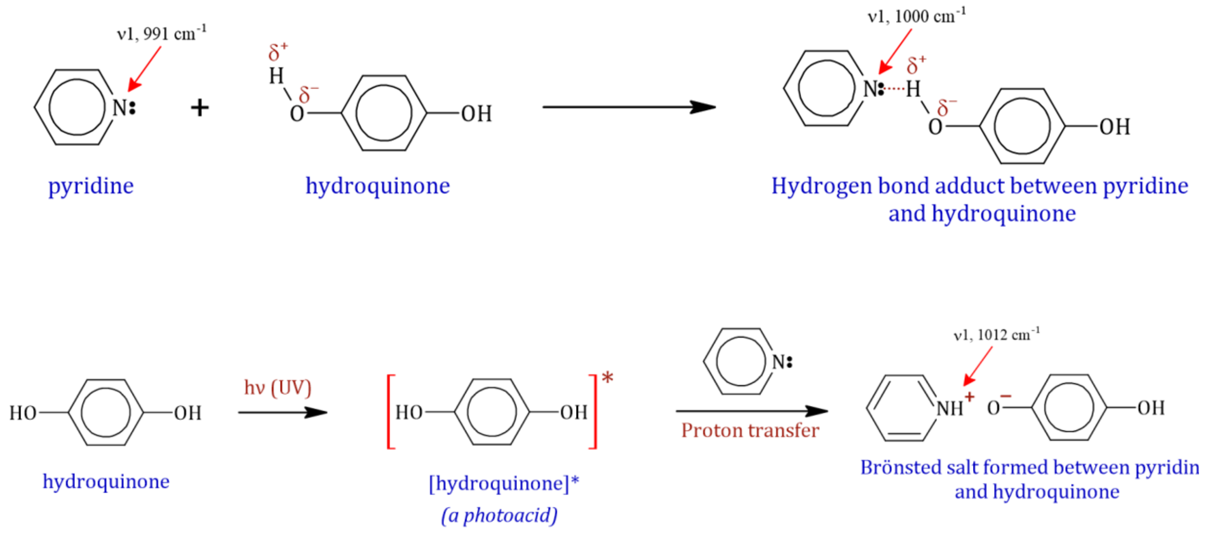


Supplementary Figure S4. Interactions of pyridine as a Lewis base with H2Q related species: hydrogen bonding with the -OH group of hydroquinone (top), or proton transfer from a hydroquinone molecule acting as a photoacid (bottom). The Raman shift at which the pyridine symmetric ring breathing band (ν1) would appear is shown for each species involved.

## S5. Hyperfine coupling constants ($A_N$ and $A_H$) in EPR spin trapping experiments with DMPO

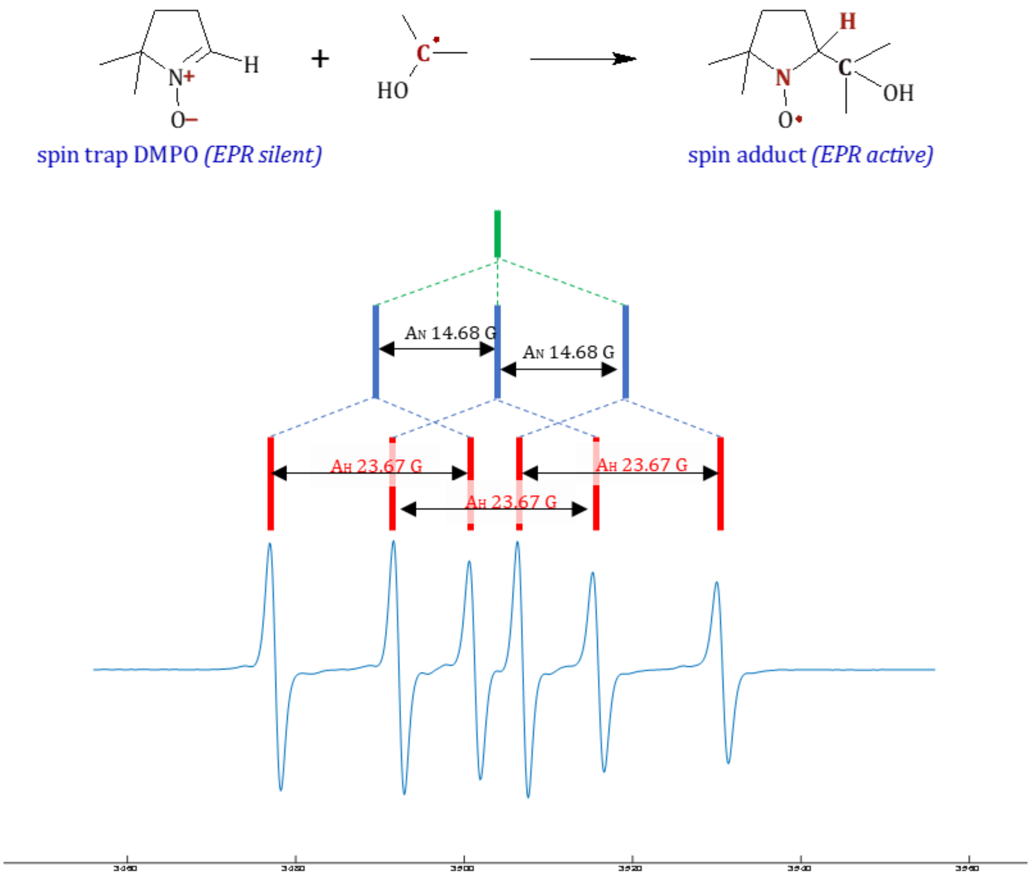


Supplementary Figure S5. Calculation of hyperfine coupling constants (AN and AH) in EPR spin trapping experiments with DMPO.

These constants show how the unpaired electron interacts with nitrogen and hydrogen nuclei. Each interaction splits the signal into 2**n**I+1 lines, where *n* is the number of equivalent nuclei and I is their nuclear spin.

Therefore, the interaction with one $^{14}N$ (I= 1) nitrogen results in a splitting of the radical signal into three lines. Each of these three lines splits further into two because of the interaction with the $^{1}H$ atom (I=1/2).

## S6. Details of the UV lamp used in the process

Reactions were carried out at room temperature under UV light (UV Spotlight Source Lightningcure™ L8022, Hamamatsu, Shizuoka Pref., Japan), with the light focused on the sample compartment through an optical fiber. Accessible on 04 August 2025
*https://www.hamamatsu.com/jp/en/product/light-and-radiation-sources/lamp-module_unit/spot-light-source/L9588.html*

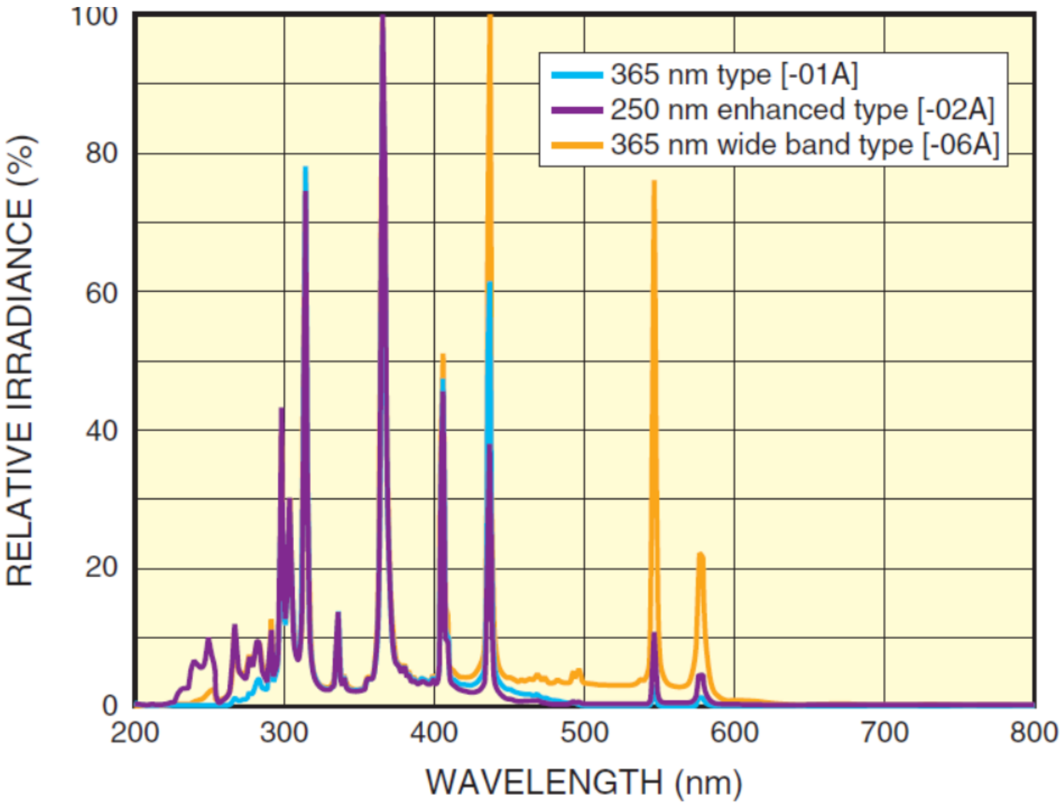


Supplementary Figure S6. UV spectral distribution of UV spotlight from Hamamatsu (365 nm-type blue trace, type [-01A]) as provided by the supplier (Hamamatsu, Shizuoka Pref., Japan).
